# Quantifying the contribution of utility cycling to population levels of physical activity: an analysis of the Active People Survey

**DOI:** 10.1093/pubmed/fdv182

**Published:** 2017-02-02

**Authors:** Glenn Stewart, Nana Kwame Anokye, Subhash Pokhrel

**Affiliations:** 1 Health Economics Research Group (HERG), Institute for Environment, Health and Societies, Brunel University London, Uxbridge, Middlesex UB83PH, UK

**Keywords:** physical activity, public health, active transport

## Abstract

**Background:**

Population levels of physical activity are far below recommendations limiting its public health benefits. Utility cycling (i.e. cycling for transport purposes) may be a means of increasing this activity. Empirical evidence quantifying the contribution of utility cycling to the population levels of physical activity is sparse.

**Methods:**

The English Active People Survey (APS) was analysed to assess the likelihood of meeting UK physical activity guidelines in those who reported utility cycling compared with those who did not. Odds ratios were adjusted for important socioeconomic confounders using a logistic regression model.

**Results:**

In the full sample, unadjusted odds ratio for meeting physical activity guidelines in favour of utility cyclists was 5.21 (95% confidence interval (CI) 4.96–5.47) and adjusted odds ratio was 4.08 (95% CI 3.88–4.29). The odds were even higher for utility cyclists in inner London [adjusted OR: 6.08 (4.07–7.86)]. The pattern was consistent regardless of the number of activities through which people met the physical activity guideline.

**Conclusion:**

Utility cycling can make a significant contribution to levels of physical activity. As an activity that can easily integrate into everyday life, utility cycling appears to be a pragmatic policy option for public health decision-makers.

## Introduction

Physical activity is essential for maximal health. It is associated with a 30% reduction in all-cause mortality as well as a reduction of between 20–40% in many long-term conditions such as cardiorespiratory, metabolic, musculo-skeletal and mental health, breast and colon cancers.^1^ It has been described by a Chief Medical Officer (CMO) of England as a ‘wonder drug’^2^ with benefits accruing most quickly to those moving from ‘no’ activity to ‘some’ activity.^1^ It is described by the Academy of Royal Colleges as a ‘miracle cure’.^3^

Despite wider acknowledgement of public health benefits of physical activity (PA) and the publication of guidelines by over 20 countries across the globe as well as by the World Health Organisation (WHO),^4^ population levels of physical activity remain low. An international survey of 122 countries using three definitions of physical activity estimated that 31.1% (95% CI 30.9–31.2) of adults (aged 15+) are physically inactive with a range of 4.7% (95% CI 4.3–5.1) in Bangladesh to 71.9% (31.0–87.2) in Malta.^5^

In the UK, the currently recommended ‘dose’ is 150 min a week moderate physical activity or 75 min intense activity (or a combination of the two) in bouts of 10 min or more for those aged 19–64 as well as improving muscle strength on 2 days a week. Older adults are similarly encouraged to undertake 150 min of physical activity a week. Further guidance is available for those aged 0–4, 5–18 and 65+.^1^ These guidelines are similar to those of the US Department of Health and Human Sciences^6^ and the World Health Organisation.^7^

It has been argued that the most acceptable means of enabling people to increase their levels of physical activity will be through activities that can be incorporated into everyday life, for example, walking or cycling rather than using motorized transport.^1^ Evidence from Northern European countries where up to 27% of journeys are be undertaken by cycle^8^ indicates that cycling, for a purpose upon completion of the journey, as opposed to a trip that is undertaken for the purpose of the journey in itself  ^9^ may be one means by which population levels of physical activity can be increased.^10^ ‘Utility cycling’—as an independent activity or done in conjunction with sport—therefore may be one way of gaining the benefits of increased levels of physical activity across the population while also avoiding the external costs of motorized transport including pollution, noise, congestion and road traffic injuries.^11^ This may have important implications for increasing levels of physical activity in countries such as England where participation in sport does not seem to be increasing. The annual Active People Survey (APS),^12^ the largest sports participation survey in England, has consistently found that population participation in sport and/or active recreation has remained remarkably stable with only between 34.6 and 36.9% of adults reporting undertaking at least 1 × 30 min of moderate intensity sport per week between October 2006 and March 2015.^13^

There is a paucity of empirical data showing the extent to which utility cycling could increase population levels of physical activity. The aim of this study therefore was to quantify the contribution that cycling for utilitarian purposes might make to the likelihood of meeting physical activity recommendations at a population level, either in conjunction with sport or as an activity in its own right. The findings are expected to provide insights as to where next to increase population levels of physical activity.

## Methods

### Design

An empirical analysis with a cross-sectional design was implemented to establish whether there were significant differences in the recommended levels of physical activity between those who did utility cycling and those who did not. Participants were recorded doing or not doing utility cycling in the past 4 weeks and mapped to the levels of physical activity they reported for the same period. By allowing for any other correlations that might have existed in the system, this design allowed us to quantify the ‘net’ contribution of utility cycling to population levels of physical activity.

### Data

Data were sourced from the Active People's Survey (APS),^12^ an annual cross-sectional survey on sport and active recreation in England conducted for Sport England, a non-departmental public body sponsored by the Department for Culture, Media and Sport (DCMS). The APS is a random digit dialling (RDD) survey weighted to be representative of each reporting geographical area (Local Authority, County Council, London Borough, Government region). In many parts of England, there are two tiers of local government: county councils and district, borough or city councils. In other parts, there is just one tier of local government comprising unitary authorities, London boroughs and metropolitan boroughs. Each of these has different responsibilities and powers.^14^

Within each geographical area, the survey was weighted by age, gender, ethnicity, socioeconomic classification, household size and working status.^15^ The survey was originally of people aged 16+ but was extended in the final quarter of APS 6 (October 2011–October 2012) to include those aged 14+. Interviews are distributed evenly over each 12-month period. APS 7 drew on nationally representative sample of 165 191 people (aged 14+) with fieldwork taking place between 15th October 2012 and 14th October 2013. The survey is designed to achieve a minimum of 500 interviews in most Local Authorities. The person in the household interviewed is selected through Computer-Aided Telephone Interviewing software that randomly selects either the telephone responder or any other adult in the household for the survey. Response rates to the survey have consistently been ∼25%.

For practical purposes, residents in institutions (armed forces barracks, student halls of residence, hospitals, care homes, etc.) are excluded, and it is recognized that mobile-only households (∼15% in 2011) are also excluded. From October 2012 to October 2013, ∼1000 interviews were undertaken using a RDD mobile phone survey and a shortened version of the APS questionnaire. Compared with landline respondents mobile phone respondents were more likely to be male, younger and from non-white ethnic groups than landline responders. No systematic difference was found between landline and combined landline–mobile results for once a week or once a month participation for the 10 largest sports nor once a week or three times a week participation for all sports. No physical measurements were undertaken.

For our analysis, we used APS 7 data with a final sample size of 165 191 of which 66 962 (40.5%) were male and 98 229 female (59.5%).

### Outcome variable

Meeting recommended levels of physical activity was the outcome variable. Two alternative specifications of this variable was used: (i) meeting Chief Medical Officer (CMO) guidelines of 600 min moderate to vigorous physical activity in the past month either through any number of activities; (ii) that through one activity only (i.e. sport or utility cycling or utility walking).

### Explanatory variables

The main explanatory variable was ‘utility cycling’. It was measured as a binary variable that took a value of 1 if the respondent had undertaken utility cycling (either solely or in addition to other activities) and 0, otherwise. Utility cycling was defined as cycling for purposes other than for the purposes of health, recreation, training or competition.

In the APS, people who reported general cycling but reported zero days of cycling for the purposes of health, recreation, training or competition were considered as doing ‘utility cycling’. The survey question was framed as: ‘I would now like you to think about any cycling you may have done. Please include any casual cycling in your local area, any cycling in the countryside or on cycling routes, cycling to or from work or any competitive cycling. In the last four weeks, that is since [^INSERT DATE^] have you done any cycling?’ The response categories were 1 (Yes), 2 (No) and 3 (Don't know). In addition to walking and cycling, APS participants were asked about all activities they had done in the last 4 weeks whether for competition, training, receiving tuition, socially, casually or for health and fitness. A list of included sports/activities is included in Supplementary data, Appendix 1. The format for sporting and active recreational questions was as follows: ‘I have already asked you about walking and cycling. I would now like to ask you about other types of sport and recreational physical activity you may have done. Please think about all the activities you did, in the last four weeks, whether for competition, training or receiving tuition, socially, casually or for health and fitness, but do not include any teaching, coaching or refereeing you may have done. So thinking about the last four weeks, that is since [^INSERT DATE^], did you do any sporting or recreational physical activity?’ Response categories were (1) Yes, (2) No and (3) Interviewers do not read out. Code if respondent has stated they are severely disabled and do no activity. Code only as a last resort if respondent is frustrated or unhappy with activity, and (4) Don't know. These two sets of questions together let us create a utility cycling variable as specified above.

Other explanatory variables included were age (16–34, 35–54, 55+), gender (male/female), ethnicity (White, Asian, Black, Chinese, Mixed, Other), National Statistics Socio-Economic Classification (NS-SEC) grades (1–4, 5–8, 9),^16^ number of children in the household (0, ≥1) and region (North East, North West, Yorkshire and the Humber, West Midlands, East Midlands, East of England, South West, South East and London). The choice of these sociodemographic and other variables was informed by previous research in this area.^17^

### Statistical analysis

Means (standard deviation—SD) and proportions were calculated for continuous data and categorical data as appropriate. We used the *χ*^2^ and Fischer's exact tests to examine whether missing data occurred completely at random. All variables were categorical and we used the indicator method to adjust for missing data (i.e. item non-response was included in the omitted category).^18^

A logistic regression model was used to estimate the likelihood of meeting recommended levels of physical activity for participants who undertook utility cycling compared with sports and exercise adjusting for other covariates. Two sets of logistic regression models for each specification of the outcome variable were fitted. First unadjusted model allowed bivariate analysis examining the relationship between meeting the recommended level and each of the individual explanatory variables separately. Second, an adjusted analysis allowed a multivariate analysis in which all explanatory variables were included in the same model. The analysis was repeated for the sample of inner and outer London residents separately to see whether the likelihood of meeting the recommended levels of physical activity might differ.

Goodness of fit was evaluated using quintiles rather than the usual deciles as the Hosmer–Lemeshow test is less likely to over- or under-predict observations in large data sets with a smaller number of quantiles.^19^ Specification errors were tested in all models using the linktest. Both unadjusted and adjusted odds ratios (AORs) were computed for each independent variable. The threshold for statistical significance was set at ≤5% in all analyses. Analyses were undertaken using Stata SE 12.^20^

## Results

### Sample characteristics

Table 1 summarizes the characteristics of the sample. Of the 165 191 people who took part in APS7, 66, 962 (40.5%) were males. The majority of respondents were aged 55+ (51%), white (91%), of NS-SEC categories 1–4 (56%) and without children in the household (72%). Just over 20% participants in the sample were from South East of England, whereas 4% came from the North East. Age had the highest number of missing observations (55%) and ethnicity the least (21%).

**Table 1:** Sample characteristics

*Characteristics*	*Number (%)*	*Met physical activity recommendations (%)*
Gender		
Male	66 962 (40.54)	41.87
Female	98 229 (59.46)	30.27
Age		
16–34	25 693 (15.55)	55.21
35–54	53 784 (32.56)	40.22
55+	83 622 (50.62)	25.54
Missing	2092 (1.27)	
Ethnicity		
White	149 998 (90.80)	34.79
Mixed	1609 (0.97)	47.67
Asian	4733 (2.87)	35.73
Black	3439 (2.08)	37.22
Other	748 (0.45)	38.77
Chinese	198 (0.12)	40.91
Missing	4466 (2.70)	
Socioeconomic status		
NS SEC 1–4	92 921 (56.25)	35.40
NS SEC 5–8	52 023 (31.49)	31.90
NS SEC 9	18 155 (10.99)	37.92
Missing	2092 (1.27)	
Number of children in household		
None	119 178 (72.15)	32.09
One or more	38 570 (23.35)	42.87
Don't know/missing/ refusal	7443 (4.51)	
Region		
North East	6084 (3.68)	34.07
North West	20 286 (12.28)	33.84
Yorkshire	10 684 (6.47)	34.93
West Midlands	15 219 (9.21)	32.45
East Midlands	20 343 (12.31)	33.55
East	23 893 (14.46)	34.68
South West	18 893 (11.10)	33.90
South East	33 998 (20.58)	35.87
London	16 355 (9.90)	

Compared with the national average London respondents were younger, more ethnically diverse and slightly more likely to have children (Table 2).

**Table 2:** London, inner London and outer London sample characteristics

*Characteristics*	*London*		*Inner London*		*Outer London*	
	*Number (%)*	*Met physical activity recommendations (%)*	*Number (%)*	*Met physical activity recommendations (%)*	*Number (%)*	*Met physical activity recommendations (%)*
Gender						
Male	6394 (39.10)	3200 (50.05)	2345 (38.43)	1236 (52.71)	4028 (39.57)	1954 (48.51)
Female	9961 (60.90)	4416 (41.62)	3757 (61.57)	1751 (46.61)	6151 (60.43)	2382 (38.73)
Age						
16–34	3809 (23.29)	2221 (58.31)	1537 (25.19)	956 (62.20)	2240 (22.01)	1254 (55.98)
35–54	5974 (36.53)	3017 (50.50)	2285 (37.45)	1257 (55.01)	3.670 (36.05)	1766 (48.12)
55+	6353 (38.84)	1961 (30.87)	2218 (36.35)	740 (33.36)	4116 (40.44)	1208 (29.35)
Missing	219 (1.34)		62 (1.02)		153 (1.50)	
Ethnicity						
White	10 999 (67.25)	5044 (45.86)	4037 (66.16)	2047 (51.37)	6861 (67.40)	2938 (42.82)
Mixed	521 (3.19)	238 (54.32)	237 (3.88)	125 (52.74)	287 (2.82)	158 (55.05)
Asian	1694 (10.36)	711 (41.9)	411 (6.74)	194 (47.20)	1250 (12.28)	506 (40.48)
Black	1929 (11.79)	781 (40.49)	879 (14.41)	347 (39.48)	1069 (10.50)	445 (41.63)
Other	319 (1.95)	140 (43.89)	151 (2.47)	73 (48.34)	183 (1.80)	74 (40.44)
Chinese	69 (0.42)	37 (53.62)	30 (0.49)	17 (56.67)	37 (0.36)	18 (48.65)
Missing	824 (5.04)					
Socioeconomic status						
NS SEC 1–4	9518 (58.20)	4502 (47.30)	3601 (59.01)	1889 (52.46)	5825 (57.23)	2574 (44.19)
NS SEC 5–8	4099 (25.06)	1523 (37.16)	1440 (23.60)	556 (38.61)	2647 (26.00)	967 (36.53)
NS SEC 9	2519 (15.40)	1174 (46.61)	999 (16.37)	508 (50.85)	1554 (15.27	687 (44.21)
Missing	219 (1.34)					
Number of children in household						
None	10 928 (66.82)	4645 (42.51)	4254 (69.71)	2011 (47.27)	6638 (65.21)	2635 (39.70)
One or more	4554 (27.84)	2300 (50.51)	1511 (24.76)	824 (54.53)	2938 (29.36)	1454 (48.65)
Don't know/missing/ refusal	873 (5.34)		337 (5.52)		552 (5.42)	

A total of 112 816 (68.29%) participants reported undertaking at least 1 min of physical activity in the past 4 weeks. In London, 12 625 (77.19%) reported the same. Approximately a quarter of respondents reported at least 1 min of utility walking and no other activity, <1% utility cycling and no other activity and just <20% one-minute sport with no other activity (Table 3).

**Table 3:** Sample undertaking at least 1 min physical activity through various activities – England, London, inner and outer London

	*England number (%)*	*London number (%)*	*Inner London number (%)*	*Outer London number (%)*
At least 1 min utility walking (no sport, no utility cycling)	38 783 (23.48)	4672 (28.57)	1755 (28.76)	2893 (28.42)
At least 1 min utility cycling (no utility walking, no sport)	1130 (0.68)	77 (0.47%)	38 (0.62)	39 (0.38)
At least 1 min sport (no utility walking, no utility cycling)	31 325 (18.96)	2264 (13.84)	777 (12.73)	1481 (14.55)
At least 1 min utility walking or utility cycling or sport	112 816 (68.29)	12 625 (77.19)	4808 (78.79)	7619 (74.85)

### Contribution of utility cycling to meeting the recommended level

Table 4 shows the unadjusted and adjusted odds of meeting the recommended level of participation through any number of activities (Model 1) and through one activity only (Model 2) for the explanatory variables. Individuals who undertook utility cycling had higher odds of meeting the recommended levels of physical activity compared with those who did not undertake utility cycling (AOR = 4.08, *P* < 0.001 in Model 1 and AOR = 2.73, *P* < 0.001 in Model 2). Utility cyclists were therefore ∼3–4 times as likely to meet recommended levels of physical activity as those who were not, after allowing for other correlates.

**Table 4:** Unadjusted and adjusted odds of meeting physical activity guidelines

*Independent variables*	*Model 1 (meeting guidelines regardless of any number of activities)*		*Model 2 (meeting guidelines through one activity only)*	
	*Unadjusted odds ratio (95% confidence interval)*	*Adjusted odds ratio (95% confidence interval)*	*Unadjusted odds ratio (95% confidence interval)*	*Adjusted odds ratio (95% confidence interval)*
Utility cycling				
No utility cycling	1.00	1.00	1.00	1.00
Utility cycling	5.21 (4.96–5.47)***	4.08 (3.88–4.29)***	3.56 (3.40–3.72)**	2.73 (2.61–2.86)***
Age				
16–34	1.00	1.00	1.00	1.00
35–54	0.59 (0.58–0.61)	0.59 (0.57–0.61)***	0.62 (0.60–0.64)***	0.62 (0.60–0.64)***
55+	0.29 (0.28–0.30)	0.30 (0.29–0.31)***	0.32 (0.31–0.32)***	0.33 (0.32–0.34)***
Gender				
Male	1.00	1.00	1.00	1.00
Female	0.61 (0.60–0.63)	0.65 (0.64–0.66)***	0.60 (0.59–0.62) ***	0.63 (0.62–0.65)***
Ethnicity				
White	1.00	1.00	1.00	1.00
Mixed ethnicity	1.78 (1.62–1.97)***	1.11 (1.01–1.24)**	1.71 (1.55–1.88)**	1.12 (1.01–1.24)**
Asian	1.08 (1.02–1.14)**	0.67 (0.62–0.71)***	1.04 (0.98–1.11)	0.67 (0.63–0.72)***
Black	1.10 (1.03–1.18)**	0.71 (0.66–0.77)***	1.11 (1.04–1.19)**	0.75 (0.70–0.81)***
Other	1.20 (1.04–1.39)**	0.76 (0.66–0.89)***	1.19 (1.02–1.38)**	0.79 (0.68–0.93)***
Chinese	1.35 (1.01–1.78)**	0.82 (0.61–1.10)	1.30 (0.98–1.72)	0.83 (0.62–1.11)
Socioeconomic status				
NS-SEC 1–4	1.00	1.00	1.00	1.00
NS-SEC 5–8	0.81 (0.80–0.83)***	0.82 (0.80–0.83)***	0.85(0.84–0.87)***	0.86 (0.84–0.88)***
NS-SEC 9	1.09 (1.06–1.13)***	0.97 (0.93–1.01)	1.11 (1.08–1.15)***	0.99 (0.95–1.03)
Children				
No children	1.00	1.00	1.00	1.00
Having children	1.69 (1.65–1.73)***	1.06 (1.04–1.10)***	1.59 (1.55–1.63)***	1.04 (1.01–1.07)**
Region				
North East	1.00	1.00	1.00	1.00
North West	0.99 (0.93–1.05)	0.98 (0.92–1.04)	0.99 (0.93–1.05)	0.97 (0.92–1.04)
Yorkshire	1.03 (0.97–1.10)	1.00 (0.94–1.08)	1.04 (0.97–1.11)	1.02 (0.95–1.09)
West Midlands	0.94 (0.89–1.00)	0.93 (0.87–0.99)**	0.93 (0.87–0.99)**	0.92 (0.85–0.98)**
East Midlands	0.98 (0.93–1.04)	0.95 (0.90–1.02)	0.98(0.92–1.04)	0.95 (0.90–1.02)
East	1.04 (0.99–1.11)	0.97 (0.90–1.03)	1.03(0.97–1.09)	0.97 (0.91–1.03)
South West	1.01 (0.95–1.07)	0.98 (0.92–1.05)	0.99 (0.93–1.05)	0.98 (0.92–1.04)
South East	1.10 (1.04–1.17)**	1.04 (0.98–1.10)	1.08 (1.02–1.15)	1.03 (0.97–1.09)
London	1.41 (1.33–1.50)***	1.28 (1.20–1.36)***	1.32 (1.24–1.41)***	1.21 (1.13–1.29)***

**P ≤ 0.05.

***P ≤ 0.01.

Meeting guidelines was associated with being younger, male, of higher socioeconomic position, having children, being from London and being of mixed ethnicity though all other ethnicities were less likely than those of white ethnicity to meet guidelines.

In inner and outer London, utility cycling was associated with, respectively, 6 and 5 times greater odds of meeting physical activity guidelines compared with no utility cycling. Other variables in outer London had the same effect as nationally in predicting the likelihood of meeting recommended guidelines. In inner London, however, gender did not predict the likelihood of meeting recommended levels (Table 5).

**Table 5:** Unadjusted and adjusted odds ratios for meeting physical activity guidelines regardless of any number of activities in inner and outer London

*Independent variables*	*Inner London*		*Outer London*	
	*Unadjusted odds ratio (95% confidence interval)*	*Adjusted odds ratio (95% confidence interval)*	*Unadjusted odds ratio (95% confidence interval)*	*Adjusted odds ratio (95% confidence interval)*
Utility cycling				
No utility cycling	1.00	1.00	1.00	1.00
Utility cycling	7.93 (6.17–10.18)***	6.08 (4.07–7.86)***	6.62 (5.30–8.27)***	5.26 (4.19–6.61)***
Age				
16–34	1.00	1.00	1.00	1.00
35–54	0.74 (0.65–0.85)***	0.69 (0.60–0.80)***	0.73 (0.66–0.81)***	0.66 (0.59–0.74)***
55+	0.30 (0.26–0.35)***	0.28 (0.24–0.33)***	0.33 (0.29–0.36)***	0.30 (0.26–0.34)***
Gender				
Male	1.00	1.00	1.00	1.00
Female	0.78 (0.71–0.87)***	0.91 (0.81–1.01)	0.67 (0.62–0.73)***	0.73 (0.67–0.79)***
Ethnicity				
White	1.00	1.00	1.00	1.00
Mixed ethnicity	1.05 (0.81–1.37)	0.82 (0.62–1.08)	1.64 (1.29–2.07)***	1.20 (0.94–1.54)
Asian	0.85 (0.69–1.03)	0.63(0.51–0.79)***	0.91 (0.80–1.03)	0.65 (0.58–0.75)***
Black	0.62 (0.53–0.71)***	0.54 (0.46–0.64)***	0.95 (0.84–1.08)	0.73 (0.63–0.84)***
Other	0.89 (0.64–1.23)	0.67 (0.47–0.95)**	0.91 (0.67–1.22)	0.68 (0.49–0.93)**
Chinese	1.24 (0.60–2.55)	0.86 (0.40–1.82)	1.26 (0.66–2.41)	0.84 (0.43–1.64)
Socioeconomic status				
NS-SEC 1–4	1.00	1.00	1.00	1.00
NS-SEC 5–8	0.57 (0.50–0.65)***	0.70 (0.61–0.80)***	0.73 (0.66–0.80)***	0.73 (0.66–0.81)***
NS-SEC 9	0.94 (0.81–1.08)	0.93 (0.79–1.12)	1.00 (0.89–1.12)	0.89 (0.78–1.02)
Children				
No children	1.00	1.00	1.00	1.00
Having children	1.34 (1.19–1.51)***	0.99 (0.86–1.13)	1.43 (1.31–1.57)***	1.00 (0.90–1.11)

**P ≤ 0.05.

***P ≤ 0.01.

Census data indicate that the local authorities with the highest rates of commuter cycling are Cambridge, Oxford, the Isles of Scilly, Hackney, York, Gosport, Islington, Norwich, Kingston upon Hull and Lambeth.^21^ On further analysis of the sample covering those top 10 commuter cycling areas only, the odds ratio in favour of utility cycling for meeting physical activity guidelines was 4.90 (95% CI 4.03–5.96).

## Discussion

This paper sought to quantify the contribution that cycling for utilitarian purposes might make to population levels of physical activity. This is in the context of marked differences in the use of cycling as a means of transport even across countries where car ownership is high such as the UK, the USA and the Netherlands^22^ and significant and rising costs of health care due to long-term conditions (LTCs).^23^

The APS is the largest survey of sport and active recreation undertaken in Europe.^24^ Analysis of this survey indicates that those who undertake utility cycling have odds of meeting physical activity four times as much as that of those who do not. In inner London, these odds could be up to six times. In the UK, it is estimated that long-term conditions account for some 70% of the NHS budget but also that 42.9% of the working population of 26.5 million has a commuting journey of under 5 km,^25^ considerably less than the 8 km cited by the BMA that a ‘person can easily cover’.^26^ Meeting physical activity recommendations is correlated with a reduction of all-cause of 30% and most long-term conditions by between 20–40%.^1^ The policy implications of our findings are profound for both public health and transport sectors. The Department for Transport has reported that schemes that encourage walking and cycling have a cost–benefit ratio of 5.62 : 1, far above the threshold considered to be of ‘high value’ of 4 : 1. Following Chief Medical Officer's recommendation of doubling the distance walked and a 8-fold increase in cycling and a doubling of distances walked, Jarret *et al.* have already estimated that active travel would save the NHS £17 billion within 20 years.^27^ Collaboration between the Treasury, Public Health England and the Department for Transport may therefore be warranted to ensure value for money through appropriate investments in transport infrastructure.^28^

It is perhaps unsurprising to find that those with the highest odds of utility cycling were young males. Young males are traditionally the most risk-tolerant section of society^29^ and therefore might be expected to cycle more where the risks of cycling are higher than in European countries where the cycling infrastructure is more developed and the risks associated with cycling are far less. For example, in the Netherlands, Germany and Denmark women cycle almost as much as men and rates only decline slightly with age.^22^

Having children in the household was associated with slightly increased odds of meeting guidelines (OR = 1.06, 95% CI 1.04–1.10). Unfortunately, the data set did not include information on car ownership for us to rule out any correlation with both having children and cycle use.

The only region in which the odds ratio of meeting guidelines was statistically significant was London in which efforts have been made to improve the cycling infrastructure through cycling super highways and bike-hire scheme.^30^ In inner London, there was no statistical difference between the genders. This stands in contrast to previous analysis of national census data 2001–11 which found no increase in the proportion of females commuting by bicycle.^31^

In contrast to the above utility cycling was more common in NS-SEC 1–4 than NS-SEC 5–8 but with no significant difference in those in NS-SEC 9. Intuitively, those on a lower income have relatively more to gain from utilitarian cycling, even if only from a financial perspective. The unadjusted odds ratio indicated significantly more utilitarian cycling in NS-SEC 9, but this disappeared after adjusting for confounding factors. This indicates that cycling in England is still predominantly male, white and affluent—signifying that its potential to reduce inequalities is not being realised, in itself part of the mission of Public Health England^32^ and statutory guidance to for all Local Authorities and Clinical Commissioning Groups through their Health and Wellbeing Strategies.^33^

High cycling prevalence countries have shown cycling for a purpose is an activity that can be integrated into everyday life and reduce the external costs of motorized transport.^22^ In this context, our findings strongly support promotion of utility cycling as an attractive option to public health decision-makers in England and perhaps elsewhere too. How public health policies could/should incorporate promotion of utility cycling is beyond the remit of this study. However, our recent systematic review^34^ suggested that individual or group-based interventions^35,36^ as well as wider environmental interventions^37–39^ could increase commuter cycling. In that review, environmental interventions were found to have small but positive effects in much larger but more difficult to define populations. These two studies together can provide public health decision-makers with robust data needed to work out various policy options to promote utility cycling.

This study has a few noteworthy limitations. Given cross-sectional data, any finding here cannot be taken as causal. Utility cycling was defined to cover cycling for utilitarian purposes either solely or in addition to other activities—there was no information to isolate the two. It may arguably be that utility cycling was predominantly used as a means of transport to sports or leisure facilities. However, this is thought unlikely as data available for London suggests that some 27% of population meet physical activity recommendations through active travel alone.^40^ Unfortunately, the data set did not include information on car ownership for us to rule out any correlation with both having children and cycle use.

## Conclusion

People who undertook utility cycling were four times as likely to meet current physical activity recommendations as those who did not undertake such cycling, even after controlling for other underlying population characteristics. In inner London where investments in cycle infrastructure have taken place, this likelihood appears to rise to six times. As utility cycling has a tremendous potential to both increase population levels of physical activity and reduce the external costs of motorized transport, promotion of utility cycling therefore appears to be a pragmatic policy option to public health decision-makers.

## Supplementary material

Supplementary material is available at *PUBMED* online.

## Supplementary Material

Supplementary Data
