# Supplementary material for: Quantifying the contribution of utility cycling to population levels of physical activity: an analysis of the Active People Survey
Source: J Public Health (Oxf). 2017 Feb 2;38(4):644–52. doi: 10.1093/pubmed/fdv182 (PMC6088795; doi:10.1093/pubmed/fdv182)
Supplement: Supplementary Data [file utility_cycling_submitted_to_jph_26th_sept_2015_appendix_1.doc.docx]

**Appendix 1: Activities included in Active People Survey 7**

| Abseiling | Bocce | Boules | Climbing - rock | Diving - deep water / free | Football (outdoors) – small sided (e.g. 5-a-side) |
| --- | --- | --- | --- | --- | --- |
| Aerobics | Bmx | Bowls | Climbing - rope | Dodge ball | Frisby / Frisbee |
| Aikaido | Bobsleigh | Bowls - Crown green | Coasteering | Drag racing | Futsal |
| Air chair | Basketball – Outdoors | Bowls - Flat green indoor | Conditioning activities / circuit training | Dragon boat racing | Gaelic football |
| American football | Baton twirling | Bowls - Flat green outdoor | Cricket | Exercise bike / spinning class | Gaelic sports |
| Aquafit / aquacise / aqua  aerobics | Biathlon | Bowls - Short mat | Cricket - other | Exercise machine /running machine / treadmill | Gliding |
| Archery | Bicycle polo | Boxercise | Cricket (Indoors) | Extreme adventure | Goalball |
| Arm wrestling | Boccia | Boxing | Cricket (Indoors) - nets /practice | Fencing | Go karting |
| Athletics - field | Body attack | Boxing | Cricket (Outdoor) - nets /practice | Fishing - Coarse | Golf - Driving Range |
| Athletics - track | Body balance | Bungee jumping / heli-bungee jumping / para bungee | Cricket (Outdoors) - match | Fishing - Game | Golf - full course |
| Australian rules football | Body boarding | Camogie | Cross training | Fishing - sea | Golf - Putting |
| Auto cross | Body building | Canoe polo | Curling | Fives - eton | Golf - Short course / Par 3 / Pitch and Putt |
| Autotest | Body combat / cardio kick | Canoeing | Cycling | Fives - rubgy | Gym |
| Backpacking | Body jam | Canyoning | Cycling - downhill riding / gravity riding | Floorball | Gymnastics |
| Badminton – Indoor | Body pump | Caving / pot holing | Cycling - stunt riding | Football (indoors) | Handball |
| Badminton – Outdoor | Body step | Cheerleading | Cyclo-cross | Football (Indoors) – small sided (e.g. 5-a-side) | Hang-gliding |
| Baseball | Body vive | Climbing - ice | Dance exercise | Football (outdoors) – 11-aside | Harness racing |
| Basketball – Indoors | Bouldering | Climbing - indoor | Deck bowls | Football (outdoors) - Other | Health and fitness |
| High wire | Ju-jitsu | Mine exploration | Nordic (previously Telemark | Rambling | Sailing – Yacht Racing (inc.Multihull) |
| Highland games | Kabaddi | Modern pentathlon | Octopush | RAQUETBALL (American version NOT played on standard squash court) | Sandboarding / sand boarding |
| Hill climb | Karate | Motor racing | Orienteering | Road racing (motors) | Sea level traversing |
| Hill trekking | Karting | Motor sprints | Paintball | Roller blading / roller skating | Self defence |
| Hockey - Field - indoor | Kayaking | Motorcycling - drag/sprint | Parachuting | Rope coursing | Sepak takraw |
| Hockey - Field - outdoor | Kayaking - whitewater | Motorcycling - enduro | Paragliding | Rounders | Shinty |
| Hockey - roller | Keepfit / keep fit / sit ups | Motorcycling - motocross | Parakarting | Rowing - Outdoor / Water based | Shooting |
| Hockey - street | Kendo | Motorcycling - off road | Parascending | ROWING MACHINE / Indoor Rowing | Skateboarding |
| Hockey - underwater | Kho-kho | Motorcycling - rallying | Petanque | Rugby – other | Skating - in-line |
| Hockey - ice | Kick boxing / thai boxing | Motorcycling - sidecar racing | Pilates | Rugby Union | Ski flying |
| Horse riding | Kite surfing | Motorcycling - super | Polo | Running - | Skiing |
| Horse riding - three day eventing | Koozahngal | Motorcycling - track racing | Polocrosse | Running - fell | Ski-ing - barefoot snow |
| Hovering | Korfball | Motorcycling - trail riding | Power kiting | Running - road | Ski-ing - barefoot water |
| Hurling | Lacrosse | Motorcycling - trials riding | Powerboat racing | Running - track | Ski-ing - extreme |
| Ice skating | LEGS, BUMS and TUMS | Mountain biking | Powerlifting | Running - ultra marathon | Ski-ing - free |
| Irish handball | Life saving | Mountain boarding | Press ups | Sailing – Dinghy Racing (inc. Multihull) | Ski-ing - grass or dry ski slope |
| Jam-alai | Luge | Mountain walking | Quoits | Sailing – Dinghy Cruising (inc.Multihull) | Ski-ing – mono |
| Jet ski-ing | Luge - street | Mountaineering | RACKETBALL (played on standard Squash Court) | Sailing - ice | Ski-ing - parachute |
| Jogging | Martial arts | Mountaineering - high altitude | Rafting | Sailing – keelboat cruising | Ski-ing - ribbing |
| Judo - Contact | Martial arts – Chinese | Netball - indoor | Rally cross | Sailing – keelboat racing | Ski-ing - speed |
| Judo - Non-contact | Medau | Netball - outdoor | Rallying | Sailing – Yacht Cruising (inc.Multihull) | Skipping |

| Skittles | Swimming - open water | Volleyball - outdoors | Wrestling - olympic freestyle |  |  |
| --- | --- | --- | --- | --- | --- |
| Sky diving | Swimming / diving [indoors] | Wake boarding | Wrestling - olympic grecoroman |  |  |
| Sky surfing | Swimming / diving [outdoors] | Walking | Wrestling - westmoreland |  |  |
| Snomobile racing | Table tennis – indoor | Walking - cliff | Yachting - ice |  |  |
| Snorkelling | Table tennis - outdoor | Walking - gorge | Yachting – land |  |  |
| Snow mountain bike racing | Taekwando | Walking - hill walking | Yoga |  |  |
| Snowboarding | Tai chi | Water polo | Zumba |  |  |
| Snowsport | Tang soo do | Waterskiing |  |  |  |
| Soaring | Tchoukball | Weightlifting |  |  |  |
| Softball | Tennis - indoor | Wheelchair sports - archery |  |  |  |
| Sombo | Tennis - outdoor | Wheelchair sports - basketball |  |  |  |
| Speed biking | Tenpin bowling | Wheelchair sports - fishing |  |  |  |
| Speedway | Tobogganing | Wheelchair sports - Rugby |  |  |  |
| Sportsboats | Trampolining | Wheelchair sports – table tennis |  |  |  |
| Squash | TRAMPOLINING - in garden | Wheelchair sports - Tennis - indoor |  |  |  |
| Step machine | Trials racing | Wheelchair sports - Tennis - outdoor |  |  |  |
| Stool ball | Triathlon | Windsurfing or boardsailing |  |  |  |
| Sub aqua / scuba diving /scuba diving | Trifoiling | Wrestling - beach |  |  |  |
| Super-modified shovel racing | Trotting | Wrestling - grappling |  |  |  |
| Surf life saving | Tug of war | Wrestling - cornish |  |  |  |
| Surfing | Ultimate frisbee | Wrestling – Cumberland |  |  |  |
| Swimming - deep water | Volleyball - indoors | Wrestling - Lancashire or 'Catch as Catch Can' |  |  |  |
